# Clinical enrollment assay to detect preexisting neutralizing antibodies to AAV6 with demonstrated transgene expression in gene therapy trials

**DOI:** 10.1038/s41434-022-00353-2

**Published:** 2022-07-01

**Authors:** Liching Cao, Annemarie Ledeboer, Yonghua Pan, Yanmei Lu, Kathleen Meyer

**Affiliations:** Sangamo Therapeutics, Inc, Brisbane, CA 94005 USA

**Keywords:** Immunological techniques, Gene therapy, Gene therapy

## Abstract

Recombinant adeno-associated virus (AAV) vectors are the leading platform for gene delivery for a variety of clinical applications. Patients with preexisting antibodies to AAV are currently excluded from most AAV gene therapy trials to avoid vector neutralization and ensure response to therapy. Anti-AAV neutralizing antibodies (NAbs) are typically assessed by in vitro cell-based transduction inhibition (TI) assays. However, clinical relevance of the determined enrollment cutoff and the inherent variability of a cell-based assay present challenges for use as an enrollment screening test. Here, we describe an enrollment cutoff that was clinically validated and strategies to overcome assay challenges to enable long-term stable performance. A validated anti-AAV6 cell-based TI assay was used to support clinical enrollment across multiple investigational gene therapies and to evaluate AAV6 seroprevalence in healthy and disease populations. The clinical enrollment cutoff was determined statistically using samples collected from healthy donors, applying a 0.1% false error rate with the inclusion of a minimum significant ratio (MSR) metric and in consideration of results from in vivo mouse passive transfer studies. Our strategy for long-term monitoring and control of assay performance employed plate quality control samples flanking the predefined cutoff. An approach using donor samples was implemented to bridge different lots of critical reagents without the need to redefine the cutoff.

## Introduction

Gene therapy delivery via recombinant adeno-associated virus (AAV) vectors holds promise for treating patients with inherited or acquired genetic diseases. Significant progress was made with the approval of two AAV-based therapies in the United States, Luxturna® in 2017 and Zolgensma® in 2019. There are many challenges associated with the clinical development of AAV-based gene therapies including the presence of preexisting antibodies to AAV capsid proteins. Serological studies show that healthy humans develop humoral immunity against AAV capsids due to natural consequences of exposure to the wild-type AAV [1]. The first clinical gene therapy study for hemophilia B using recombinant AAV2 vector to deliver the factor 9 (*F9*) gene via systemic administration showed that low titers (titer < 1:2 to 1:11 per test method used) of preexisting anti-AAV2 neutralizing antibody (NAb) completely neutralized doses of AAV2 and resulted in <1% FIX plasma levels in treated subjects [1, 2]. Thereafter, NAb status to AAV has been widely used as an exclusion criterion for patient enrollment in AAV-based clinical studies to ensure potential treatment benefits [1–5]. In the same study, subjects with similar anti-AAV2 NAb titers treated at a higher AAV2 dose did produce measurable plasma FIX levels, demonstrating that low levels of neutralization may be overcome by higher treatment doses [2]. The antibody titer range impacting efficacy and the correlation between antibody titer and effective treatment doses, however, are still unknown, and need to be determined empirically in clinical studies [3].

Both total antibody (TAb) binding immunoassays and cell-based NAb (also known as transduction inhibition (TI)) assays have been used in clinical studies to evaluate preexisting antibodies to AAV capsid for enrollment purposes [6, 7]. A TAb assay, using either a direct coat or bridging binding assay format, is more robust, simpler in design, less variable, and easier to monitor operationally. A functional cell-based TI assay has the advantage of detecting neutralization activity by directly measuring the impact on AAV transduction, however, this assay is more challenging to manage as an enrollment assay. TI assays detect total AAV neutralizing activity including NAbs as well as non-antibody factors in the blood, and the non-antibody factors remain a small percentage of the neutralizing activity response [7, 8]. The TI assay is also more challenging for long-term use due to higher variability of a cell-based assay. Likewise, maintenance of cell lines, AAV reporter, and quality controls are important reagents and factors that can impact the performance of a cell-based TI assay. Monitoring the impact of critical reagents on long-term assay performance is key to ensuring accurate and precise data. Lot-to-lot variability of AAV reporter gene titer, full-versus-empty AAV capsids, and the negative control pool can impact the assay performance and the suitability of using the same predefined cutoff for enrollment. Understanding the source of assay variability and implementing a strategy to control and monitor the assay are critical to ensure performance consistency over time. Studies have shown that TI and TAb assay methods have relatively good concordance [7, 8].

Another approach for assessing anti-AAV antibody activity is a mouse passive transfer neutralization activity assay. Mice are first intravenously (IV) administered human serum samples followed by AAV to assess the ability of preexisting antibodies in human serum to neutralize AAV-mediated transduction and transgene expression in mouse liver [9, 10]. This approach is cumbersome, time-consuming, and difficult to implement as a screening test for clinical studies but is a helpful tool to aid the understanding of anti-AAV antibody impact in vivo during assay cutoff determination [11].

In this report, we describe a validated cell-based AAV6 TI assay with the cutoff determined statistically using healthy donor samples in conjunction with in vivo mouse passive transfer assay data. Common assay monitoring approaches for cell-based TI assays utilize only a negative control and low and/or high-quality controls. This method does not address assay drift caused by high assay variability observed in a cell-based TI assay. To address this challenge, a strategy of implementing controls flanking the predefined cutoff was used for assay monitoring. Additionally, an approach to bridge different lots of critical reagents due to depletion or expiration of reagents without the need to redefine the cutoff was implemented to ensure long-term use of the assay. This well-controlled assay was used to evaluate seroprevalence in healthy donors and patients, which supported clinical trial enrollment for multiple investigational gene therapy products.

## Materials and methods

### Human serum samples

Individual human serum samples were purchased from BioIVT (Westbury, NY, USA), Discovery Life Sciences (Los Osos, CA, USA), and Golden West Biologicals (Temecula, CA, USA). Serum from hemophilia A and hemophilia B donors were purchased through custom collection from HRF, Inc (Raleigh, NC, USA). Donors varied in ethnicity (Black, White, Hispanic, Asian), ages, and geographical locations in the USA. Serum from hemophilia A and hemophilia B patients were collected from consenting subjects in Sangamo Therapeutics-sponsored clinical trials. The trials were conducted according to the principles of the Declaration of Helsinki. All subjects provided written informed consent prior to inclusion in the study. The Institutional Review Board (IRB)/Independent Ethics Committee for each site and Central IRB approved the protocols. An additional 49 serum samples from hemophilia B patients in the United Kingdom (UK) were collected during a seroprevalence study conducted at the University Hospital Southampton NHS Foundation Trust with informed consent from patients with hemophilia B registered at six UK Hemophilia Comprehensive Care Centers (S. Boyce, I. James, S. Rangarajan, et al. manuscript in preparation). The study was reviewed and supported by the Proportionate Review Sub-Committee of the East Midlands – Derby Research Ethics Committee, study number 18/EM/0313.

### AAV6 luciferase

The AAV6 vector construct containing a *Photinus pyralis* firefly luciferase reporter gene under control of the cytomegalovirus (CMV) promoter was produced in insect Sf9 cells at Virovek (Hayward, CA, USA) and formulated in buffer consisting of phosphate-buffered saline and 0.001% Pluronic F-68. AAV titers were determined by quantitative polymerase chain reaction (qPCR). Full and empty capsid ratio was determined for applicable lot using high-performance liquid chromatography [12].

### Quality controls

Negative control serum (NCS) was generated by pooling serum from individuals with tested luciferase responses comparable to the measured luciferase responses in the cell culture media (normalized ratio ranged from 0.9 to 1.3). Pooled positive human serum from donors and a mouse monoclonal anti-AAV6 antibody (ADK6) from Progen (Wayne, PA, USA) were both used to generate quality control (QC) samples for the AAV6 transduction inhibition assay. For controls made using pooled positive human serum, QC1, QC2, QC3 were prepared by spiking the pooled positive control serum into the pooled NCS at different ratios. For controls made using ADK6, QC1, QC2, QC3 were prepared by spiking ADK6 at 3.5, 1.5, and 0.9 µg/mL, respectively, into the pooled NCS. These controls were aliquoted into single-use vials and stored at −65 °C to −90 °C. Three determinations of each control (in duplicate) are assessed on every plate during sample testing. All analyses were performed using data normalized to NCS and presented as normalized response (NR). QC1, QC2, and QC3 were designed to have approximate NR of < 0.1, 0.25, 0.45, respectively with QC1 and QC2 below the clinical cutoff of 0.34 and QC3 above the clinical cutoff.

### AAV6 TI assay

The cell-based transduction inhibition assay utilized human U-87 MG (glioma cell line) HTB-14 (ATCC, Manassas, VA, USA) and an AAV6-CMV-luciferase vector construct as reporter. U-87 MG cells from the working cell bank were cultured in U-87 MG growth media containing minimum essential media (MEM) (Gibco, Grand Island, NY, USA) with 10% fetal bovine serum (HyClone, Logan, UT, USA), 1% Sodium Pyruvate (Gibco, Grand Island, NY, USA), and 1% Penicillin-Streptomycin (Lonza, Morristown, NJ, USA). Viable U-87 MG cells were seeded at 20,000 cells per well (100 µL per well) in white opaque 96-well plates. On the following day, QCs (positive and negative controls) and samples were initially diluted fivefold in U-87 MG dilution media (U-87 MG growth media with 1% bovine serum albumin), followed by another twofold dilution of U-87 MG dilution media containing AAV6-CMV-luciferase to yield a 10-fold dilution prior to incubation with cells. QC samples were analyzed at minimum required dilution of 1:10 (MRD 10) and clinical samples were analyzed at MRD 10 followed by four serial 2.5-fold dilutions, at 10% final serum prior to incubation with cells. Data from MRD 10 were used for reporting and the remaining dilutions were for information purposes only. Multiplicity of infection (MOI) of AAV6-CMV-luciferase used in the assay is lot-specific. Each new lot was bridged prior to use through partial validation including the evaluation of positive control titration curves, QCs precision, and a minimum 30 healthy donors to evaluate concordance in test results. MOI ranged from 4e4 to 1e5 across three lots were tested and bridged. Diluted samples and AAV6-CMV-luciferase (1:1 mix at 75 µL each to reach 150 µL in total volume) were incubated at 37 °C for 30–40 min. Following incubation, all media from the plated U-87 MG cells were removed via aspiration. 50 µL of U-87 MG growth media was added to all wells and 50 µL of AAV6-CMV-luciferase/sample or QCs mix was added to the plate in duplicate, bringing final volume to 100 µL. Plates were incubated for 24 h (±2 h) in a humidified 37 °C incubator, 5% CO_2_. The following day, plates were removed from the incubator and incubated at room temperature for 10–15 min. 100 µL of One-Glo reagent/buffer mixture (Promega, Sunnyvale, CA, USA) was added to each well, incubated at room temperature for 5–30 min and read via luminescence using BioTek Synergy 2 (Agilent Technologies, Santa Clara, CA, USA) plate reader.

Neutralizing antibodies and non-antibody factors against the viral capsid reduce the ability of AAV6 to transduce the cell and thereby result in lower luciferase expression. On each plate, luminescent signals from test samples are normalized to mean signal of wells transduced with AAV6-CMV-luciferase in the presence of NCS. A reduction in luciferase activity following incubation with serum dilutions indicates the presence of anti-AAV6 neutralizing activity.

### AAV6 in vivo passive transfer mouse study

All animal studies were conducted at Pacific BioLabs (Hercules, CA, USA) using 79-week-old-male C57BL/6 mice. The studies were performed in compliance with all applicable sections of the Final Rules of the Animal Welfare Act Regulations (9 CFR 1–3), the Public Health Service Policy on Humane Care and Use of Laboratory Animals, the Guide for the Care and Use of Laboratory Animals, and the guidelines of the Pacific BioLabs Institutional Animal Care and Use Committee (IACUC).

Human serum samples (100 or 200 µL), either a pool or from individual donors, were administered IV to naïve C57BL/6 mice (*n* = 5–10/group), followed 2 h later by IV delivery of 200 µL AAV6 encoding human Factor 9 (*hF9)* cDNA (Virovek, Hayward, CA, USA) at a dose level of 6e10 vg/mouse. Preexisting neutralizing activity to AAV6 capsid in the serum is anticipated to impact in vivo transduction of hepatocytes and subsequent expression of the *hF9* transgene.

Endpoints in these studies included plasma hFIX levels, which represent successful hepatocyte transduction and transgene expression. hFIX concentration in the plasma was measured by ELISA using VisuLize FIX^®^ antigen kit according to the manufacturer procedure (Affinity Biologicals, Ancaster, ON, Canada).

### Cutoff determination

Cutoff determination was performed by B2S Life Sciences (Franklin, IN, USA). All statistical analyses were completed using R (R version 3.3.1, 2016–06–21), JMP (Version 13; SAS Institute, Inc., Cary, NC, USA), and Microsoft Excel 2016. The logarithm base 10 transformed NR values from individual subject samples were used to estimate overall variability and intra-run analytical variability. Statistical methods used for the calculation of cutoff were consistent with procedures recommended by refs. [13] and [14] when applied to immunoassay designs described by ref. [13]. A total of 312 NR values generated from three independent experiments were included in the evaluation and six were identified as analytical outliers using a linear mixed-effects Analysis of Variance (ANOVA) model leaving 306 values for cutoff calculations. The ANOVA model included fixed effects of analyst, gender, and ethnicity and random effects for subject, run within analyst, and residual. Estimates for the parametric and nonparametric cutoff at 0.1%, 1%, and 5% error rates were determined using the log-transformed values. A parametric method with Tukey’s biweight procedure was used to calculate robust estimates of the mean and standard deviation (SD) of all log-transformed ratios [15]. The parametric cutoff calculation with the corresponding error rate was calculated as

$$Parametric\;cutoff = 10^{\left[ {Biweight\;Mean + Biweight\;SD \ast t_{ \propto ,n - 1}} \right]}$$

where *t*_α_, *n*−1 is the α percentile of the t-distribution with degrees of freedom equal to the number of log-transformed NR values minus 1. The nonparametric cutoff calculation with the corresponding error rate was determined by the calculation of the corresponding empirical percentile for the log-transformed NR values followed by an inverse log transformation.

Cell-based assays tend to have higher variability both due to inherent biological variability and operator variability. A statistical analysis was conducted to determine how best to compare a subject test result to a fixed cutoff value. To statistically capture this variability, the MSR [16–19] approach was used to calculate the response range for each calculated cutoff, taking into account intra-run variability. To determine the MSR, after the removal of outliers, the SD of the remaining log-transformed values was calculated using restricted maximum likelihood estimates from the mixed-effects model [14]. The intra-run SD was estimated as the square root of the residual variance from the ANOVA model. The overall SD was 0.094. The MSR of 1.27 was calculated using the formula:

$${{{{{{{\mathrm{MSR}}}}}}}} = 10^{[2 \ast {{{{{{{\mathrm{intra - run}}}}}}}}\;{{{{{{{\mathrm{SD}}}}}}}}]}.$$

The estimated response range for the corresponding false error rate was established by multiplying the cutoff estimate by 1.27 to define the upper end of the range, and the lower end of the range was determined by dividing the cutoff estimate by 1.27.

## Results

### Cell-based AAV6 TI method validation

Anti-AAV6 NAbs in human sera were detected using the in vitro AAV6 TI assay. This cell-based assay utilized the U-87 MG human glioma cell line and an AAV6 vector encoding a firefly luciferase reporter gene. The method was validated according to industry white papers [13, 14] and FDA immunogenicity guidance [20] as a guide at a contract research organization (BioAgilytix, Durham, NC, USA) under Good Laboratory Practice compliance with knowledge of Good Clinical Practice. Validation parameters included cutoff determination (detailed description in Statistical Cutoff Determination and Methods sections), precision, relative sensitivity, selectivity, specificity, and stability. Initially, the method was validated using a positive control of pooled human serum with strong transduction inhibition. A mouse monoclonal anti-AAV6 antibody ADK6 (Progen) was later included in partial validation when the reagent became commercially available. QC samples (QC1, QC2, QC3) were prepared by spiking the pooled positive control serum or ADK6 into the pooled NCS at different dilutions or concentrations, respectively. All analyses were performed using data normalized to NCS and presented as NR. Overall summary of the method validation parameters and assay performance are provided in Table 1. The relative assay sensitivity determined using ADK6 was 1.18 µg/mL at the clinical cutoff of 0.34. ADK8 (Progen, Wayne, PA, USA), an antibody specific to AAV8 serotype, did not yield signal which demonstrated assay specificity. For controls, three QCs using both pooled positive human serum and ADK6 were evaluated. QC1 which has a NR < 0.1 was designed to represent high seropositive samples. QC2 and QC3 were later designed to flank the clinical cutoff to monitor long-term assay performance. QCs generated using pooled positive human serum and ADK6 had overall intra- and inter-assay precision less than 25% coefficient of variation (CV) except for QC1 where CV acceptance was not applicable due to strong inhibition of high positive sample resulting in very low luciferase response. Post validation, CV of 25% was used as acceptance for all duplicate wells including overall NCS signal except samples with NR response <0.1 during sample testing. For clinical sample analysis plate acceptance, three sets of each QC level analyzed in duplicate wells were included at MRD 10. QC levels should yield NR in rank order of QC1 < QC2 < QC3, QC1 should have NR < 0.1, and at least two out of three determinations of each QC2 and QC3 should meet the rank order.

**Table 1:** Method validation.

Validation parameters	Validation results
Minimum Required Dilution	1:10
Clinical cutoff (NR)	0.34
Relative sensitivity using mouse monoclonal ADK6	1.18 µg/mL
Intra-assay precision (ADK6)	
QC 1 (3.5 µg/mL)	QC 1: NR 0.010; CV 32.1%
QC 2 (1.5 µg/mL)	QC 2: NR 0.279; CV 13.4%
QC 3 (0.9 µg/mL)	QC 3: NR 0.451; CV 9.3%
Intra-assay precision (pooled PS)	
QC 1 (5-fold pooled PS to NCS)	QC 1: NR 0.026; CV 9.2%
QC 2 (10-fold pooled PS to NCS)	QC 2: NR 0.205; CV 6.6%
QC 3 (14-fold pooled PS to NCS)	QC 3: NR 0.389; CV 5.9%
Inter-assay precision (ADK6)	
QC 1 (3.5 µg/mL)	QC 1: NR 0.015; CV 7.4%
QC 2 (1.5 µg/mL)	QC 2: NR 0.327; CV 14.3%
QC 3 (0.9 µg/mL)	QC 3: NR 0.409; CV 5.1%
Inter-assay precision (pooled PS)	
QC 1 (5-fold pooled PS to NCS)	QC 1: NR 0.027; CV 17.8%
QC 2 (10-fold pooled PS to NCS)	QC 2: NR 0.215; CV 21.2%
QC 3 (14-fold pooled PS to NCS)	QC 3: NR 0.359; CV 12.3%
Selectivity	ADK6 spike into 10 seronegative donors or unspike
	ADK6 at 3.5 µg/mL: 10/10 tested positive
	ADK6 at 0 µg/mL: 10/10 tested negative
Matrix Interference	No interference with hemolysis up to 400 mg/dL
Specificity	ADK8 spiked seronegative serum (NCS)
	NR with ADK8 at 3.5 µg/mL = 0.970
	NR with ADK8 at 0.9 µg/mL = 1.07
Short-term stability	Room temperature: up to tested 4 h
	2–8 °C: up to tested 24 h
	Freeze/thaw: 6 cycles

*CV* coefficient of variation, *NR* normalized response, *QC* quality control, *PS* positive serum, *NCS* negative control serum.

### Statistical cutoff determination

**Fig. 1:**
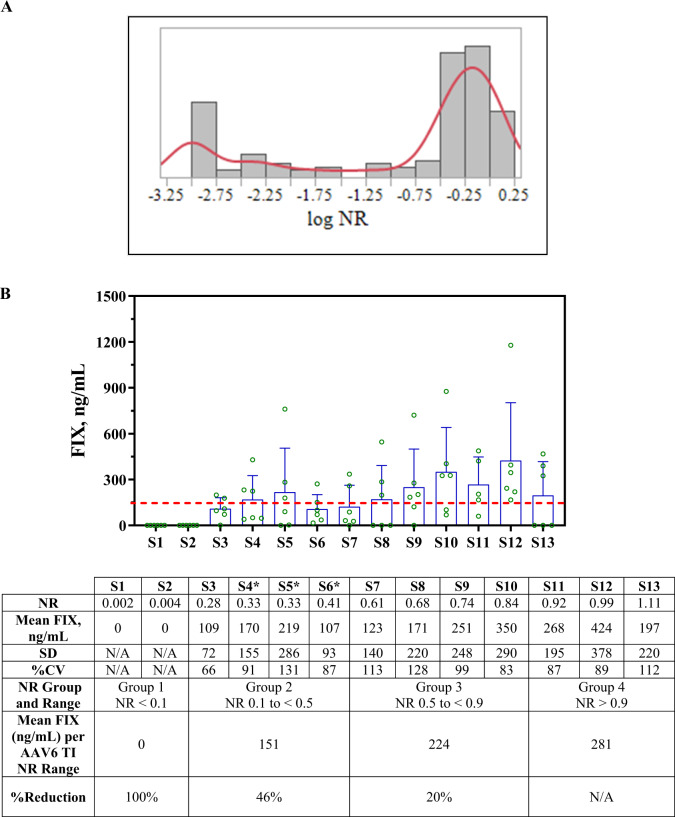
Donors response in TI assay and in vivo mouse passive transfer study. **A** 158 donors were evaluated in the AAV6 TI assay. Histogram was evaluated using JMP 14.2.0. Each bar represents a log NR bin size of 0.25 on the *x*-axis with number of donors in *y*-axis. Red line represents a smooth curve fit of data set (**B**) A total of 13 individual human serum samples, with AAV6 TI assay results ranging from 0.002 to 1.11, were tested in three separate mouse passive transfer studies. C57BL/6 mice (6 mice per donor except S11 which only has 5) were administered serum (200 µL) and AAV6 *hF9* cDNA (200 µL) at 6e10 vg/mouse via IV injection on Study Day 0. hFIX levels in plasma were measured by ELISA at Day 7 (donor with *) or Day 10 post-dosing. Each bar represents the mean plasma FIX levels from tested animals with each animal represented by an open circle, error bars indicate SD; dashed horizontal line at 150 ng/mL FIX indicates efficacious level (3% of FIX activity in healthy individuals) per literature. % Reduction in FIX level was calculated based on three NR groups (Group 1 NR < 0.1, Group 2 NR 0.1 to <0.5, and Group 3 NR 0.5 to <0.9) relative to the mean FIX level from animals treated with sera with NR > 0.9 (Group 4).

Human serum samples from 158 healthy donors were evaluated using the AAV6 TI assay to characterize data distribution. Data from each donor were normalized to the mean value of AAV6 luciferase activity incubated with cell culture media as the NCS had not yet been identified. The histogram evaluation with a log NR bin size of 0.25 revealed a bimodal distribution (Fig. 1A). An initial conservative outlier threshold with NR of 0.7 (>30% inhibition) was used to remove potential positive samples. Fifty-two samples with NR ≥ 0.7 (≤30% inhibition) were used during method validation for cutoff determination.

The data set was statistically analyzed at B2S Life Sciences (Franklin, IN, USA) to define the cutoff. Detailed description of the statistical analysis is described in the Methods section. The cutoff determination used a linear mixed-effects ANOVA model, with fixed effects of analyst, gender, and ethnicity, and random effects for subject, run within analyst, and residual. Estimates for the parametric and nonparametric cutoff at 0.1%, 1%, and 5% error rates were determined using the log-transformed values listed in Table 2. Since cell-based assays tend to have higher variability, the MSR [16–19] commonly used in potency assays to characterize the reproducibility of potency estimates generated from a concentration-response assay was used to define a response range for each of the calculated cutoff values. This range stratifies the calculated cutoff value and provides a better estimation of the lowest cutoff value to use for subject enrollment. Within each of the defined ranges, the difference in the results is considered not statistically significant and results outside of the range can be considered as statistically significant (*p* < 0.05). The calculated MSR is 1.27 based on data from method validation as described in the “Methods” section. The estimated response range for the corresponding false error rate was established by multiplying the cutoff estimate by 1.27 to define the upper end of the range, and the lower end of the range was determined by dividing the cutoff estimate by 1.27 (Table 2). To further guide the selection of the enrollment cutoff to support clinical studies, the results of AAV6 transduction in vivo studies were considered.

**Table 2:** Cutoff determination.

False positive error rate	Cutoff type	Cutoff estimate	Range of individual sample results incorporating MSR
5%	Parametric	0.593	0.47–0.75
5%	Nonparametric	0.621	0.49–0.79
1.0%	Parametric	0.511	0.40–0.65
1.0%	Nonparametric	0.560	0.44–0.71
0.1%	Parametric	0.431	0.34–0.55

Estimates for the parametric and nonparametric cutoff at 0.1%, 1%, and 5% error rates were determined using the log-transformed values. Only parametric approach was computed for 0.1% false error rate as nonparametric cannot be computed. The range of individual sample results incorporating MSR was calculated for each cutoff estimate to by multiplying the cutoff estimate by 1.27 to define the upper end of the range and the lower end of the range was determined by dividing the cutoff estimate by 1.27.

### AAV6 in vivo passive transfer mouse study to guide clinical cutoff selection

The passive transfer mouse study was used as a tool to understand the impact of preexisting antibodies on AAV6 transduction in vivo, to guide the in vitro TI assay clinical cutoff determination. Healthy human serum samples initially tested using the in vitro TI assay with a range of NR between 0.002 to 1.11 were then evaluated in mouse passive transfer studies as described in the Methods section. Human serum was administered IV to mice, followed 2 h later by an IV injection of 6e10 vg/mouse of a rAAV6 vector encoding *hF9* cDNA under the control of a liver-specific promoter. To minimize the potential impact of anti-hFIX antibody development and confounding effects, plasma hFIX levels were measured at early time points of 7- or 10-days post-AAV administration. The results from the in vivo evaluation (Fig. 1B) showed that only relatively high levels of neutralizing activity (NR lower than 0.1) in human serum were capable of fully inhibiting hFIX expression. Though the results are highly variable, animals exposed to human serum with NR > 0.33 on average still produced levels of hFIX (>150 ng/mL) that are considered therapeutic [2, 21, 22]. The in vivo studies support the selection of the statistically determined NR of 0.34, the lower end of the defined response range incorporating the MSR, at 0.1% false error rate as the clinical cutoff. This clinical cutoff (NR of 0.34) was therefore used in hemophilia A and B clinical studies for subject enrollment.

### Clinical validation of the determined AAV6 TI cutoff

A validated cell-based AAV6 TI assay with cutoff of 0.34 calculated statistically using healthy donor samples was used to screen hemophilia A patients supporting the SB-525/PF-0755480 (NCT03061201) Phase I/II study. A total of 37 subjects (a subject was screened twice and counted as one subject in this evaluation) were screened and 22 subjects (59%) with NR ≥ 0.34 passed the enrollment criteria based on preexisting antibody response to AAV6. Eleven participants were enrolled into four different cohorts with two participants each in Cohorts 1 to 3, and 5 participants in Cohort 4. Enrolled participants had NRs ranging from 0.38 to 1.11 (Table 3). Participants with the lowest and highest NR were enrolled in Cohort 4. Plasma human factor 8 (FVIII) activity increased from baseline starting in Cohort 2 and was generally dose-dependent. Participants in Cohort 4 achieved a mean FVIII activity in the normal range within 5 weeks with steady-state FVIII activity achieved by week 9 post infusion (manuscript in preparation) [23].

**Table 3:** AAV6 TI NR response range of subjects enrolled in SB-525–1603 hemophilia A study.

NR	0.34 to 0.5	>0.5–0.75	>0.75
Cohort 1 9e11 vg/kg	1	1	NS
Cohort 2 6e12 vg/kg	NS	2	NS
Cohort 3 1e13 vg/kg	NS	2	NS
Cohort 4 3e13vg/kg	2	2	1

*NR* normalized response, *NS* no subject.

### AAV6 seroprevalence and seroconversion evaluation

**Fig. 2:**
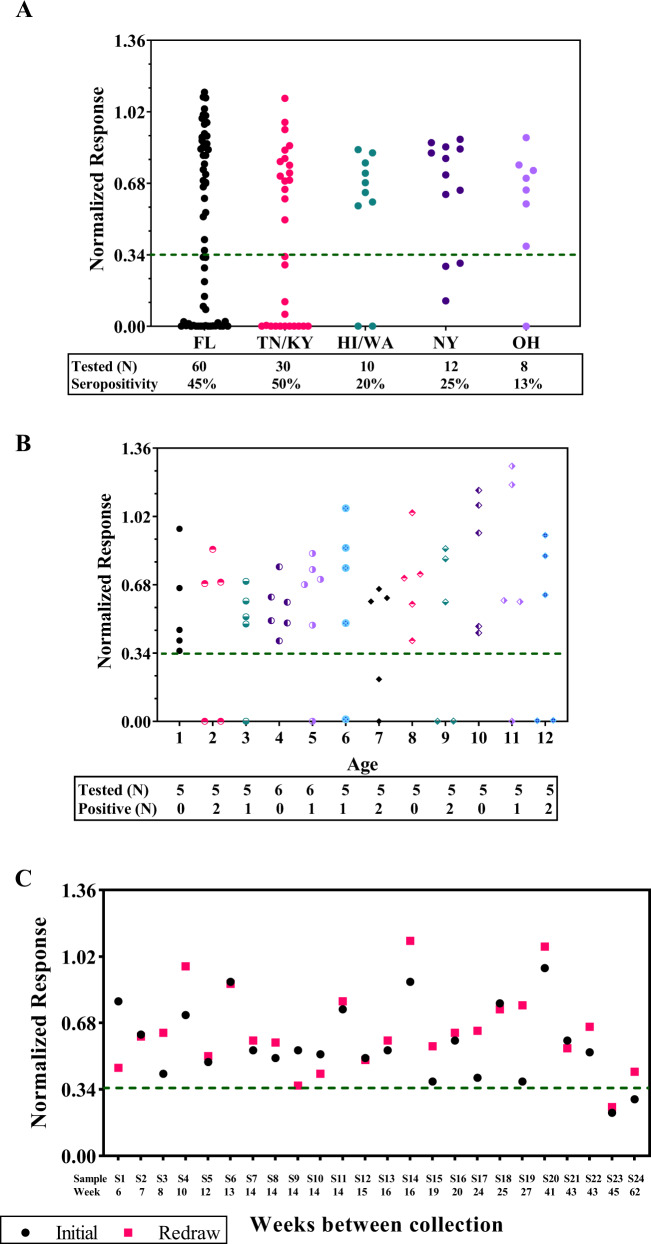
AAV seroprevalence and seroconversion. **A** Samples from a total of 120 healthy donor from different regions within the United States were evaluated. Each dot represents the normalized response (NR) from a donor. The green dashed line represents a clinical cutoff of 0.34. FL Florida, TN Tennessee, KY Kentucky, HI Hawaii, WA Washington, NY New York, OH Ohio, N Number. **B** Samples from a total of 62 pediatric donor with ages ranging from less than 1 to 12 were evaluated. Each dot represents the NR from a donor and each age group with a different symbol. The green dashed line represents a clinical cutoff of 0.34; N = Number. **C** 24 subjects across different clinical trials had blood re-drawn and reanalyzed during screening period. Black dots represent the initial analysis and magenta squares represent the analysis from a new blood collection. The green dashed line represents a clinical cutoff of 0.34.

The validated cell-based TI method was used to evaluate seroprevalence of preexisting neutralizing activity against the AAV6 capsid in both healthy and disease-state donors. Healthy donors included 120 adult (age 18 to 62) and 62 pediatric (newborn to age 12) subjects with samples collected in the United States. Out of the 120 healthy adults, 48 (40%) tested positive. Ninety donors were selected from Southern US region states (Florida (FL)/Tennessee (TN)/Kentucky (KY) states) and 30 donors from other regions (Hawaii (HI)/Washington (WA)/New York (NY)/Ohio (OH) states). Of the 48 seropositive donors, 42 (47%) donors were from the Southern states and 6 (20%) were from other regions (Fig. 2A). Sixty-two pediatric subjects with ages ranging from <1 to 12 years of age were tested. Twelve (19%) tested positive with highly positive donors detected as early as 2 years of age (Fig. 2B). The validated TI method was also used to evaluate AAV6 seroprevalence for hemophilia A and hemophilia B donors obtained from commercial sources as well as from our clinical studies with hemophilia A and hemophilia B subjects. Collectively, a total of 385 serum samples from adult and pediatric subjects were tested for seroprevalence evaluation, which ranged from 10% to 41% with an overall seropositivity of 31%, 34%, and 19% for adult and pediatric subjects, respectively (Table 4).

**Table 4:** AAV6 seroprevalence evaluation.

Population	Total Tested	<Cutoff 0.34	Seropositivity
Healthy Adults	120	48	40%
Healthy Pediatrics	62	12	19%
Hemophilia A (commercial)	56	23	41%
Hemophilia B (commercial)	52	5	10%
Hemophilia B (UK seroprevalence study)^a^	49	15	31%
Hemophilia A (SB-525–1603 study)^b^	37	15	41%
Hemophilia B (SB-FIX-1501 study)	9	3	33%
Overall	385	121	31%
Overall (adults)	323	109	34%

^a,b^Manuscript in preparation for both studies.

Often in clinical trials, preexisting anti-AAV antibody status is only valid for a certain window of time as seroconversion is a concern. Subjects need to be rescreened for enrollment eligibility if this time is exceeded. Additionally, there is interest to determine whether those with preexisting AAV neutralizing activity show a loss of activity over time. Twenty-four subjects across multiple clinical trials had blood samples re-drawn and re-analyzed. Sample collections ranged from 6 to 62 weeks apart and only one subject close to the cutoff had a change in seroprevalence result (Fig. 2C). Two positive subjects (S23 and S24) were assessed to determine whether their preexisting NAb response was reduced over time. S23 remained positive and S24 who tested positive initially, tested negative 62 weeks later. Since the NR value of S24 was close to the cutoff, assay variability may have contributed to the observed results. Overall, seroconversion rate remained relatively low (<5%) in our evaluation and aligned with published data [24].

### Assay maintenance and monitoring

To maintain the assay for long-term use, a strategy was employed to bridge new lots of critical reagents due to depletion or expiration of reagents, such as AAV6 luciferase and the NCS, without the need to redefine the cutoff.

**Fig. 3:**
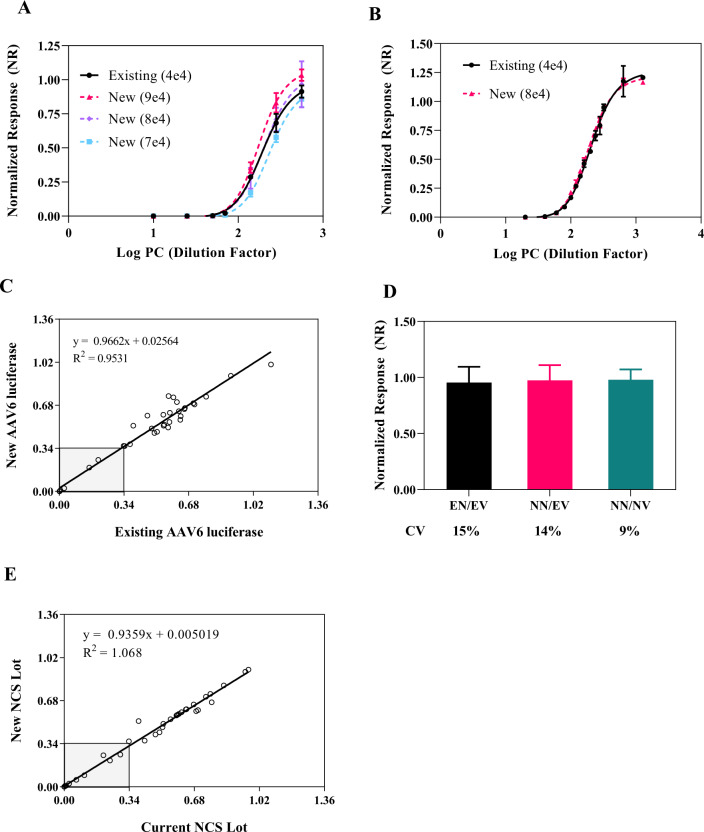
AAV6 luciferase and negative control bridging. **A** Serial dilutions of pooled serum from positive healthy donors were evaluated using the existing lot of AAV6-luciferase at 4e4 MOI (black solid circle and line) and the new lot of AAV6-luciferase at MOI of 7e4 (light blue square and dashed line), 8e4 (lavender diamond and dashed line) and 9e4 (magenta triangle and dashed line); error bars indicate the SD for the respective sample dilution and condition. **B** More refined serial dilutions of positive pooled serum were evaluated using the existing lot of AAV6-luciferase at a 4e4 MOI in two separate dilution curves (back solid circle and line) and the selected new AAV6-luciferase lot at 8e4 MOI determined from 3A (magenta triangle and dash line). **C** 30 healthy donors and 6 assay quality controls were evaluated using the existing and new AAV6-luciferase lot at 4e4 and 8e4 MOI, respectively. A linear plot (slope = 0.9662 and *R*^2^ = 0.9531) of NR response from both reagent lots shows comparable results with 100% concordance in seropositivity. Nine samples within the gray box are seropositive samples. **D** Bridging of a new negative healthy donor serum pool using the existing and new AAV6-luciferase lots (*n* = 16 samples). EN/EV existing negative/existing AAV6-luciferase (black), NN/EV new negative/existing AAV6-luciferase (magenta), NN/NV new negative/new AAV6-luciferase (green). The mean NR of the negative control in EN/EV was calculated and individual values were normalized to the mean NR to obtain %CV for EN/EV; all other conditions were normalized to mean NCS from EN/EV for %CV calculation. **E** 30 healthy donors were evaluated using the existing and new negative control serum lots. A linear plot (slope = 0.9359 and *R*^2^ = 1.068) of NR response from both reagent lots shows comparable results with 100% concordance in seropositivity. Nine samples within the gray box are seropositive samples. CV coefficient of variation, NCS negative control serum, PC positive control (pool serum), *R*^2^ R-squared.

A new lot of AAV6 luciferase would need to be bridged to the existing lot prior to use due to variability in the qPCR vector titering assay used to determine AAV6 concentration. Additionally, the differences in full/empty capsid ratio could also impact the MOI used in the assay. A 3-step approach was used to bridge different lots of AAV6 luciferase. First, to bridge the existing AAV6 luciferase (MOI of 4e4) with a new lot, various MOIs (2e4 to 1e5) of the new lot were tested against the existing lot using pooled positive serum samples by comparing the NR of each titration curve (data not shown). Subsequently, the MOIs that flanked the existing lot were further evaluated at much narrower MOI increments to determine the optimal MOI that gave comparable normalized response as compared to the existing lot. For example, a MOI of 8e4 of the new lot gave a comparable NR to the existing lot (Fig. 3A). In the second step, serial dilutions of the pooled positive control serum titration curve were further tested using existing (MOI of 4e4) and new (MOI of 8e4) lots for normalized response and half-maximal inhibitory concentrations (IC_50_) comparison. Comparable NR and IC_50_ values were obtained. The mean titer (dilution factor) at IC_50_ for the existing and new lots were 210 and 191, respectively with an overall IC_50_ of 200 with 6% CV across both lots (Fig. 3B). In step 3, 30 donors and 6 controls with various NR responses were tested at the respective MOI for each lot. 100% concordance in test results (positive versus negative) was obtained (Fig. 3C) including samples close to the cutoff of 0.34.

To bridge the NCS pooled samples using both lots of AAV6 luciferase used previously, acceptance of the normalized response to the existing NCS and assay condition was set to be within NR value of 0.9 to 1.1 and overall CV of ≤20%. For the existing AAV6 luciferase lot, the mean NR of new NCS was 0.972 and CV of 14%. With the new AAV6 luciferase lot and new NCS lot, the mean NR was 0.990 with CV of 9% (Fig. 3D). These two lots of NCS using different lots of AAV6 luciferase demonstrated comparable assay performance and concordance in donor results (Fig. 3E).

**Fig. 4:**
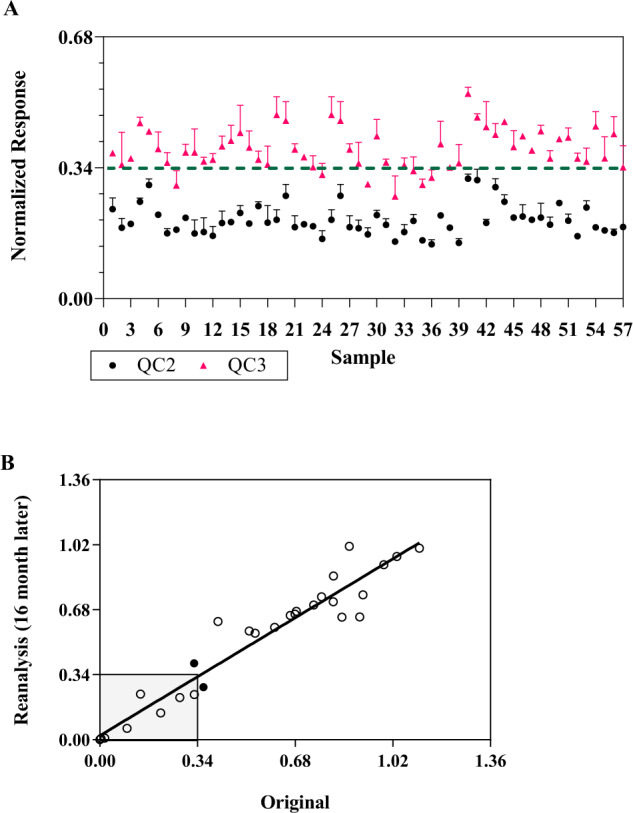
Assay monitoring using controls and donor samples. **A** Control data collected from 19 sample testing runs over 19 months. Three sets of QCs (QC 1, 2, and 3) were included during sample analysis. Each data point represents one QC analyzed in duplicate wells with mean data and standard deviation presented. Data from QC1 with NR < 0.1 is not shown. QC2 (black circle) and QC3 (magenta triangle) are designed to flank the cutoff of 0.34. All QC2 were on the positive side of the cutoff with NR between 0.1 and 0.34. For QC3, if using the acceptance criterion of only one out of three QC determinants can be below the cutoff, 18 out of 19 runs met the acceptance criterion with QC3 on the negative side of the cutoff (NR ≥ 0.34). The dashed line at 0.34 is the clinical cutoff. The collective data from long-term assay monitoring will be used to help define additional assay acceptance criteria for future implementation. **B** 30 samples were tested 16 months apart. Based on original data, *n* = 11 for positive and *n* = 19 for negative. Two discordant seropositivity samples relative to the 0.34 (93%, 91%, 95% agreement in overall, positive, negative, respectively) cutoff value are represented by a solid circle. The solid black line represents the linear regression analysis with a slope of 1.103 and *R*^2^ of 0.9307.

Typically, immunogenicity assays employ two positive QCs and one negative control. This implementation, however, is not sufficient to address the assay drift from run to run for this assay’s context of use. To better control and monitor the assay for long-term use, a strategy of using controls flanking the clinical cutoff was employed to improve the confidence in the reported results. As indicated previously, QC1 is designed to represent high positive samples, and QC2 and QC3 are designed to flank the clinical cutoff to monitor long-term assay performance. Each QC level contains three determinants run in duplicates. QC data were collected from 19 runs performed over a period of 19 months. The overall mean NRs for QC1, QC2, and QC3 were 0.0034, 0.20, and 0.38 and CVs of 48.3%, 14.8%, and 11.5%, respectively. QC2 and QC3 data are plotted in Fig. 4A. All QC2 were on the positive side of the cutoff with NR between 0.1 and 0.34. For QC3, if using the acceptance criterion of only one out of three QC determinants can be below the cutoff, 18 out of 19 runs met the acceptance criterion with QC3 on the negative side of the cutoff (NR ≥ 0.34). The collective data from long-term assay monitoring will be used to help define additional assay acceptance criteria for future implementation based on this approach of QCs flanking the cutoff to better control and monitor assay drift. In addition to QC trending, assay monitoring included using individual donors tested over time. Thirty samples tested 16 months apart were also comparable with 93% (28/30) concordance (Fig. 4B). The discordant results were from two samples close to the cutoff (NR of 0.36 vs. 0.27 and 0.33 vs. 0.40) and within the assay variability.

## Discussion

A cell-based AAV6 TI assay was developed and validated with human serum at a MRD of 1:10 to detect preexisting serum neutralization activity to AAV6 in support of patient enrollment in AAV6-based clinical trials. A statistical approach aligning with immunoassay white papers [13–15], FDA [20], and EMA [25] guidance documents was used as a guide to calculate the cutoff instead of an IC_50_ titer-based approach [6]. An initial donor response evaluation revealed a bimodal distribution in the normalized response NR values from treatment naïve subjects. One population had a high NR (presumed negative) and the other population had a low NR response (presumed positive); however, there was slight overlap of these two populations, and no definitive separation in the data to parse out one group of samples from the other. As this functional assay directly measured inhibition to AAV transduction, the cutoff was evaluated using 0.1%, 1%, and 5% false error rate. A MSR approach, commonly used to define a statistical response range was applied to further refine the cutoff. Additionally, data from the mouse passive transfer studies were considered in the clinical cutoff selection. Passive transfer studies in mice indicated only donors with relatively high levels of serum neutralizing activity (NR lower than 0.1) were capable of fully inhibiting hFIX expression. Samples with NR > 0.33 in the TI assay, although showing reduction in hFIX expression as compared to negative control serum, were capable of producing therapeutic levels of hFIX (>150 ng/mL) in some animals [2, 21, 22]. This orthogonal approach helped select the clinical cutoff for the cell-based AAV6 TI assay. This led to the decision of using NR of 0.34 as the cutoff for hemophilia A and hemophilia B clinical trials.

An important consideration is whether the cutoff is clinically meaningful, thus the determined cutoff should be confirmed in clinical trials. For the hemophilia, A SB-525–1603 Phase 1/2 study, enrolled subjects had NRs ranging from 0.38 to 1.11. A dose-dependent FVIII activity was observed with therapeutically relevant steady-state FVIII activity achieved by week 9 post-infusion in Cohort 4 (manuscript in preparation) [23]. The cutoff of 0.34 thus led to enrollment of hemophilia A subjects that responded to AAV6 gene therapy treatment. We observed about 8% of the population falls within a NR of 0.1 to 0.34 during seroprevalence evaluation. The impact of preexisting AAV6 NAbs within this slightly more positive range and resulting clinical efficacy is unknown and will require further evaluation. The method and determined clinical cutoff are well suited to support enrollment of subjects into AAV6 gene therapy clinical trials.

AAV6 seroprevalence by US geographic region was evaluated and regional differences were observed. Southern states generally had a higher seropositive rate (47%) as compared to seropositivity in other regions (20%) within the United States. AAV6 seroprevalence was also evaluated in a pediatric population (age ≤ 12) showing a 19% positive rate and detected as early as 2 years of age. These results are similar to that reported in the literature [26]. Seroprevalence for tested disease populations (hemophilia A and B) ranged from 10% to 41% with an overall mean seropositivity of 34% including healthy adult donors. Since seropositivity is highly dependent on geographic regions and disease populations, it is beneficial to perform seropositivity assessments with samples collected from geographical regions and countries where clinical studies will be conducted and from the relevant disease population, as this may facilitate prediction of enrollment eligibility during clinical trials based on preexisting AAV antibody status. Additionally, seroconversion is a concern for clinical trial enrollment and preexisting antibody response to AAV status is often only valid for weeks to months. Re-analysis of 24 available patient samples from multiple clinical trials with two sample collections ranging from 6 to 62 weeks apart indicated that the seroconversion rate for AAV6 appears to be low during this timeframe.

Clinical trial recruitment can be lengthy, especially for rare disease studies. A well-controlled method is needed to ensure the validated assay and determined cutoff can be used to support patient enrollment over a long period of time. To use this methodology as an enrollment screening test, the assay will need to be maintained with strategies in place to control and monitor the assay for long-term use. This includes maintaining a robust cell culture procedure if a single-use cryopreserved cell approach is not feasible, understanding the source of lot-to-lot variability of critical reagents such as AAV6 luciferase and assay controls, and extensive training to qualify analysts. To control and monitor the assay for long-term use, we have employed a strategy using controls flanking the clinical cutoff. This strategy further increases the confidence in assay performance and validity of test results, as the common approach using only one low positive QC close to cutoff is not adequate to monitor assay drift. Additional strategies to avoid the recalculation of the cutoff due to bridging of new critical reagent lots, such as the AAV-reporter due to depleted or expired reagents, is critical to provide uniformity across clinical data sets. A significant change in the enrollment cutoff may require retesting of clinical samples and justification will be needed if the change in the cutoff results in a change in seropositivity. We have observed different lots of AAV6 luciferase yielded different TI assay signals using the same MOI potentially due to different empty capsid content and inherent variability in the qPCR titering method, thus the MOI will typically need to be redetermined for each lot. To ensure the MOI of the new AAV6 luciferase lot does not change the assay performance, a well-thought-out bridging approach including positive control titration, QCs performance evaluation and, most importantly, inclusion of individuals with different NR values as part of the partial validation, should be evaluated to ensure concordance in test results with respect to the defined clinical cutoff.

In summary, the robust AAV6 TI assay and determined clinical cutoff described here were well suited to determine patient eligibility based on preexisting anti-AAV6 NAbs in clinical trials using AAV6 vectors for gene delivery as transgene expression was demonstrated in treated subjects. The results further demonstrated the cell-based TI assay is sufficient to evaluate preexisting antibodies to AAV6 for clinical trial enrollment, and that the approaches recommended here for long-term assay monitoring will support consistent assay performance for the length of the clinical trial.

## Data Availability

Data generated can be found within the published article.
